# Neural Plasticity in Sensorimotor Brain–Machine Interfaces

**DOI:** 10.1146/annurev-bioeng-110220-110833

**Published:** 2023-02-28

**Authors:** Maria C. Dadarlat, Ryan A. Canfield, Amy L. Orsborn

**Affiliations:** 1 Weldon School of Biomedical Engineering, Purdue University, West Lafayette, Indiana, USA;; 2 Department of Bioengineering, University of Washington, Seattle, Washington, USA; 3 Department of Electrical and Computer Engineering, University of Washington, Seattle, Washington, USA; 4 Washington National Primate Research Center, Seattle, Washington, USA

**Keywords:** brain–machine interface, sensory, motor, learning, plasticity, neural circuits

## Abstract

Brain–machine interfaces (BMIs) aim to treat sensorimotor neurological disorders by creating artificial motor and/or sensory pathways. Introducing artificial pathways creates new relationships between sensory input and motor output, which the brain must learn to gain dexterous control. This review highlights the role of learning in BMIs to restore movement and sensation,and discusses how BMI design may influence neural plasticity and performance. The close integration of plasticity in sensory and motor function influences the design of both artificial pathways and will be an essential consideration for bidirectional devices that restore both sensory and motor function.

## 1. INTRODUCTION

Our goal as humans is to interact with the world: We react to elements in our environment and act upon them via our sensory and motor systems. Injury and disease can interrupt sensorimotor function, fundamentally limiting our ability to engage. In cases where people have permanent paralysis, brain–machine interfaces (BMIs) aim to restore independence by providing artificial sensorimotor function. BMIs have restored the ability to communicate by controlling typing (1,2) or basic tasks of daily living via control of a prosthetic limb (3,4). BMIs hold tremendous promise, but improvements in performance (level of functional restoration) and robustness (consistency in performance over time and across users) are central challenges for widespread translation.

A typical BMI involves recording neural activity from a patient’s brain, transforming neural signals into motor commands, and providing sensory feedback to enable goal-directed movement and error correction. Building a BMI defines a series of computations that could be individually optimized but are interconnected as part of a closed-loop system. The tight link between sensory inputs and motor outputs in closed-loop BMIs engages innate neural mechanisms for learning, thereby changing the nature of engineering problems that must be solved to optimize BMI performance.

In this review, we describe the role of learning in closed-loop BMIs for artificial motor output or artificial sensory input. In both contexts, we restrict our focus to invasive approaches to neural measurement and modulation where significant research has explored the biological circuits involved. We also focus on topics related to the closed-loop, adaptive nature of BMIs. Insights into how neurons respond and adapt to artificial sensorimotor tasks will inform critical developments in new recording and neuromodulation technologies and noninvasive systems.

## 2. NATURAL AND ARTIFICIAL SENSORIMOTOR FUNCTION

Even a simple task like grabbing an object has many steps. We use visual information to estimate an object’s location, size, and texture. We use visual and proprioceptive information to estimate the position and movement of our arm. Finally, motor plans are made, executed, and continuously updated using incoming sensory information (5).Thus, movement, often considered a pure motor activity, is really an integrated sensorimotor computation.

Natural sensorimotor function requires that sensory and motor circuits develop through paired experience. Animals exposed to sensory information in the absence of motor commands do not learn to make precise, sensory-guided movements, as removing the link between sensory input and motor output disrupts sensorimotor function (6, 7). Paired sensorimotor experience is also needed to predict the sensory consequences of one’s own movement (8)—a computation thought to be central to motor control (9).

Relationships between sensory input and motor output are initially learned, and continually adapt. Sensorimotor flexibility allows us to navigate new environments, adapt after injuries, and even learn new skills. For instance, we can readily adapt to changes in movement kinematics imposed by a force field and shifts in the relationship between visual feedback and movement (10). This sort of adaptation requires not only plasticity in motor control but also plasticity in sensory perception (11), emphasizing that movement and sensation are intertwined functions. Given the closed-loop function of sensation and motor control in natural systems, optimizing BMIs to restore sensorimotor function requires considering the close interactions between both systems. The flexibility of closed-loop sensorimotor function also highlights the need to consider learning in BMIs.

**Figure 1:**
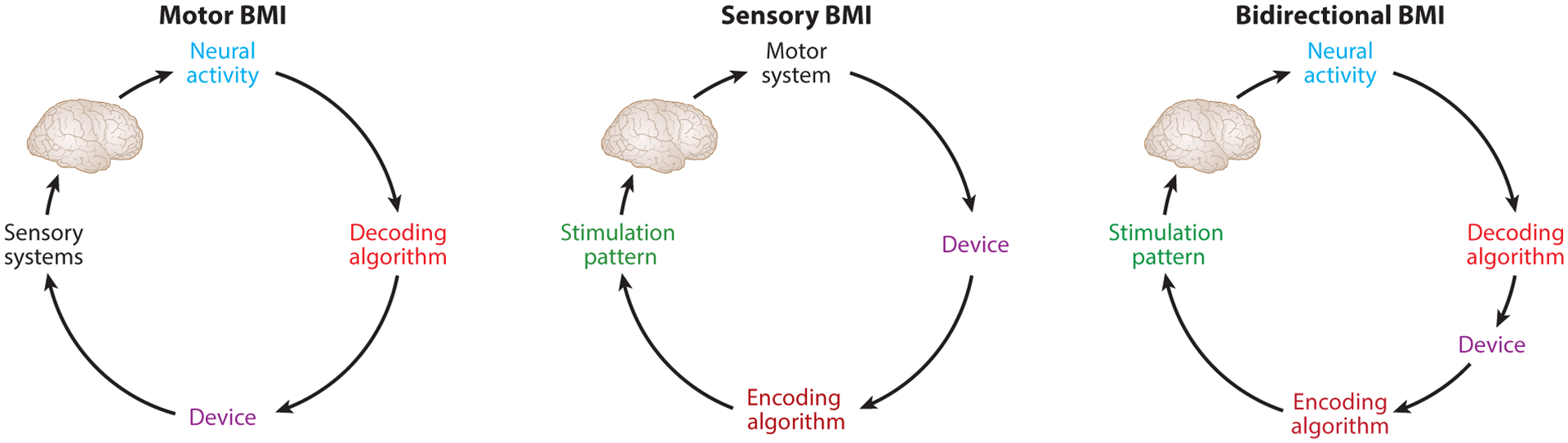
Closed-loop brain–machine interfaces (BMIs).

BMIs introduce artificial elements into sensorimotor pathways (Figure 1). Depending on the application, artificial elements may be used for motor output only (motor BMIs), sensory input only (sensory BMIs), or both (bidirectional BMIs). Importantly, all of these systems are closedloop; information flows between sensory and motor systems. All BMIs require integration of motor and sensory systems.

The artificial pathways for a BMI involve multiple components. For artificial motor output, sensors measure neural activity from portions of the brain. Measured signals are processed to generate neural features, which are inputted into an algorithm called a decoder. The decoder translates neural features into a movement command for a device. Sensory feedback of device movement ultimately closes the information loop. For artificial sensory input, a sensory stimulus from the environment is translated into a pattern of neural activation that is delivered via a stimulation device. Sensory inputs are used to guide movements, which in turn influence sensory stimuli received from the environment.

## 3. MOTOR BRAIN–MACHINE INTERFACES

Motor BMIs provide an artificial means to control movements through newly created pathways. They record neural activity from portions of natural motor circuits, translate recorded neural activity into movement commands, and send those commands to a device. Sensory feedback of movement closes the control loop. In this section, we focus on motor BMIs in which sensory feedback is provided through native pathways, which represent the majority of existing research. An example is BMI control of a computer cursor using visual feedback.

Historically, motor BMI design focused solely on maximizing the ability to predict a subject’s intended movements. For example, algorithms to map neural activity into motor commands (the decoder) are commonly chosen on the basis of predictive accuracy in data sets where subjects performed or imagined moving as neural activity was recorded. This prediction is termed open-loop because the user does not have real-time feedback of decoded movements. Improvements in open-loop predictive performance do not necessarily translate into improved performance in closed-loop BMIs (12, 13).

The difference between open-loop prediction accuracy and closed-loop BMI performance stems from the inherent integration of sensory and motor function. Creating artificial motor pathways also alters available sensory information, such as proprioception (the sense of the body’s position and movement through space), and the link between sensory and motor information. That this does not catastrophically disrupt motor performance is likely due to mechanisms that continually update our sensorimotor systems to new environments and tasks. As a result, algorithms that optimally decode neural activity offline can have minimal benefits in closed-loop systems (12).

The differences between open- and closed-loop systems change the nature of engineering problems for motor BMIs. BMI design cannot only consider prediction of motor intent—we must also consider how the full BMI sensorimotor system will be controlled and learned. Optimizing closed-loop motor BMIs will therefore require insight into the principles of sensorimotor control and learning in BMIs. In the following subsections, we summarize key observations about learning in motor BMIs and review how each element within a BMI may influence these processes.

### 3.1. Learning in Closed-Loop Motor Brain–Machine Interfaces

**Figure 2:**
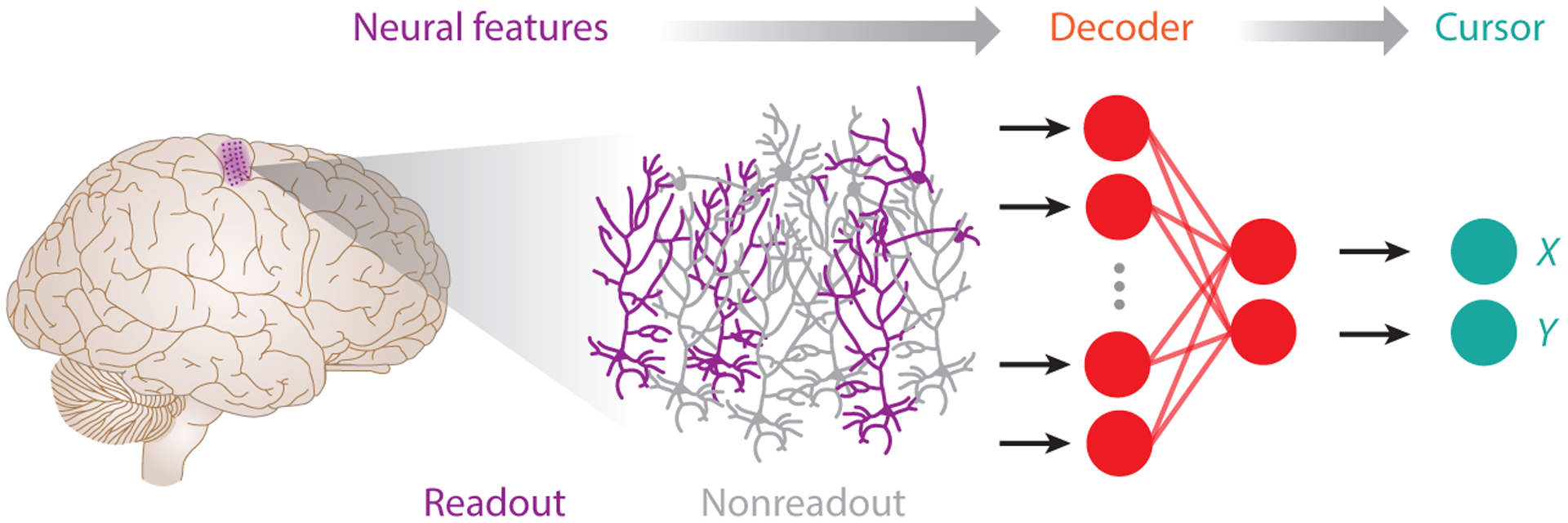
Motor brain–machine interfaces define multistep sensorimotor transformations. This illustration highlights the transformation in an example interface where the firing of 100 neurons in the primary motor cortex (M1) controls the velocity of a two-dimensional cursor through a linear decoding algorithm. Of the many brain areas that contribute to movement, we chose to measure from one (M1). Our sensors measure only a small fraction of the thousands of neurons in M1. These 100 neurons now define the motor output (behavioral readout; *purple*), while all other neurons can only indirectly contribute to movement (nonreadout; *gray*). Finally, the decoding algorithm maps a pattern of neural activity in the 100 readout neurons into a two-dimensional velocity.

BMIs present the brain with two interrelated problems (Figure 2). The brain must first generate consistent neural activity in the features chosen for BMI control (readout) and then learn how a given pattern of neural activity relates to movement. To generate patterns of neural activity specific to the readout population, the brain may have to perform some form of credit assignment (9), where movement errors are attributed to particular neurons or connections. A growing body of research has explored learning in motor BMIs. These studies highlight a rich set of phenomena related to mapping and credit assignment problems with strong similarities to those of natural sensorimotor learning (14).

Perturbing BMI decoders has shed light on how the brain adapts to altered sensorimotor maps. Much like force-field and visuomotor perturbations (9, 10), these experiments change how the activity of readout neurons relates to movement variables. Perturbations that rotate neuron– behavior relationships produce deviations in movement trajectories (15–18) or goals (19, 20) that the brain can counteract in tens to hundreds of trials. Perturbations have been applied to both single neurons (changing the direction in which a neuron moves the cursor) (15–17, 19, 20) and neural populations (changing how neural firing patterns move the cursor) (18). Behavioral adaptation to decoder perturbations resembles natural visuomotor adaptation, including the learning timescale and the presence of aftereffects following the removal of perturbations.

Neural plasticity during adaptation is distributed. Neural changes are consistent with subjects changing their aim (15, 19–21), which involves learning a new association between the target location and the action chosen but does not require altering movements themselves. Reaiming produces changes in all neurons in a population (19) but does not change the patterns of neural activity generated—only how they are associated with movement targets (21). While these global learning mechanisms dominate, if only a subset of neuron–movement relationships are perturbed, learning leads to a mixture of global and targeted changes (15–17).The presence of multiple learning mechanisms is consistent with the current understanding of computations driving visuomotor adaptation (22).

The form of decoder perturbation also influences learning. A series of experiments used decoder perturbations structured so that the experimenters could control whether the perturbation required neural populations to change their correlation structure. Mappings where neuron correlation structures do not have to change are learned within a day (18; discussed above). In contrast, perturbations where correlation structure must change require multiple days of training and lead to new neural activity patterns (23). Learning mechanisms in natural sensorimotor control are also influenced by the type of visuomotor perturbation. For instance,rotations in movement–vision relationships appear to lead to updates to an existing internal model of sensorimotor relationships, while mirror reversals lead to the creation of new models (24). Potential correspondence between natural sensorimotor learning mechanisms and observations in BMI remain to be established, but existing research clearly demonstrates the presence of multiple learning mechanisms that are flexibly deployed (14).

Another line of research explores how the brain learns initial control of a BMI. These experiments define some fixed neuron–movement relationship in a task structure that resembles de novo skill learning in natural sensorimotor systems (9, 24). These studies have found that the activities of single neurons (25, 26), neural populations (27), and local field potentials (LFPs) (28) can all be operantly conditioned. When using neuron populations for control (most comparable to the decoder perturbation studies discussed above), learning to control a new decoder mapping takes multiple days (27), and performance improves both with practice and after breaks in training (29, 30). Once learned, these mappings can be recalled and resist interference (27), paralleling natural motor skills (31).

Distributed neural plasticity underlies the acquisition of control of a new decoder. Improvements in performance coincide with the formation of stable relationships between the activity of readout neurons and movement (27). Neural patterns are variable initially, but the variance gradually decreases with learning (32, 33). Both patterns are similar to observations in natural skill acquisition (34, 35). Experiments measuring activity across multiple brain areas but using only one for the BMI readout demonstrate that neural changes are distributed across cortical and subcortical regions (28, 36–38). Blocking plasticity in the striatum can also extinguish the ability to learn a BMI decoder controlled by primary motor cortex (M1) activity (36).

BMI learning may involve credit assignment computations on long (multiday) timescales. Plasticity when learning a novel decoder leads to differential activation of readout neurons in comparison to nonreadouts, even when they are immediately next to one another (30, 39–41). Similarly, while brain areas not used for readout, such as the striatum, are needed for learning, changes in striatal neurons are targeted to those that project to readout neurons (37), consistent with plasticity targeted to a readout-specific neural circuit. Perturbing the decoder for only a subset of readout neurons produces changes specific to perturbed units that emerge over days (17). These credit assignment–related computations may be sleep dependent (30), consistent with their emergence over multiday training.

While many questions remain, these studies highlight that closed-loop BMIs engage a diverse range of learning mechanisms that parallel innate functions of natural sensorimotor systems. Learning is directly related to the sensorimotor transformations defined by the BMI system,which include all aspects of the artificial control loop from neural recording to sensory feedback. Optimizing BMI performance, then, will require considering how each element of a BMI system influences this transformation and learning computations.

### 3.2. Brain Areas

A key choice in a motor BMI is which part of the brain to record from. Differences in the computations performed by each area influence the ability to predict motor intention from neural activity. They also play different functional roles in sensorimotor learning computations (9), which may influence closed-loop performance (42).

#### 3.2.1. Motor areas.

Motor BMI research originated in M1 (25). Anatomically,M1 is positioned to contribute to motor output: It is the cortical area with the largest proportion of cells that directly project to the spinal cord (43). Its diverse inputs also include peripheral sensory afferents, highlighting contributions to sensorimotor computations (43). How M1 represents movement variables is an area of active debate (44). However, M1 activity accurately predicts a variety of kinematic (45, 46) and dynamic variables, including muscle activity (47), in open-loop systems. In consequence, M1 is often used for continuous BMIs such as controlling moment-by-moment cursor velocity.

Premotor cortical areas have also been explored for BMI control. These areas are anatomically defined as frontal regions that send projections primarily to M1; many also send projections to the spinal cord (43). Therefore, they are positioned to support preparation and execution of movements. Premotor area computations are not fully understood, but they are distinct from those of M1. For instance, dorsal premotor cortex (PMd) activity precedes that of M1, suggesting contributions to planning (48). This observation has motivated the use of premotor areas to control discrete BMI tasks, such as selecting sequential target pairs (49). However, PMd activity is also often combined with M1 for continuous control tasks (50–52).

The parietal cortex, particularly the posterior parietal cortex (PPC), is another motor area used for BMI control (53). Anatomical connections position the PPC to transform sensory inputs to motor outputs: It predominantly receives input from sensory areas and sends dense projections to frontal motor areas (43,54). Motor-related activity in the PPC plans movement goals (55) in visual coordinates (56) rather than muscle activity; as a consequence, the PPC has been used primarily for discrete BMI tasks.

#### 3.2.2. Nonmotor areas, noncortical areas, and multiple areas.

Building upon research showing that neural activity in many cortical regions can be operantly conditioned (see Section 3.1), select studies have used brain areas outside the motor system for BMI readout. For example, rodents can control an auditory pitch cursor using activity in the primary visual cortex (V1) (57), learning with the same timeline and accuracy as using M1 activity (36); this observation suggests common principles across brain areas. Interestingly, animals could learn to control a V1 BMI with the lights on or off, but could not generalize across conditions (57). Innate functions and connections between brain areas, then, likely influence learning processes and functionality. The distributed nature of motor-related signals in the brain (58), combined with the anatomical flexibility provided by learning, opens opportunities for clinical applications where people have damage to motor areas (e.g., cortical stroke) (59).

Subcortical areas have not been used for direct BMI device control. However, motivated by subcortical contributions to learning, researchers have used striatal signals to adapt cortical decoding algorithms (60). Such multiarea decoding strategies may be particularly valuable given that cortical–subcortical interactions are necessary for BMI learning (36, 57).

Leveraging multiarea activity may require additional insights into learning. Early BMI studies combined activity from many cortical areas (45). Open-loop decoding suggested that M1 was most predictive, but after closed-loop training all areas contributed to BMI movement. Alternatively, extended training with electrocorticography (ECoG) BMIs across multiple areas resulted in the consolidation of control to a small region (61). The dynamic nature of closed-loop BMIs presents challenges in a priori selection of brain regions for motor output.

#### 3.2.3. Learning differences across areas.

Few studies have quantitatively compared closed-loop BMI performance between areas. Synthesis across the literature, however, highlights important qualitative differences when BMIs are controlled with different brain areas.

Learning dynamics differ in BMIs controlled with frontal versus parietal motor areas. Studies in M1 and PMd show that the brain can learn a wide range of mappings (23, 25–27). This learning can occur within a session (18, 19) or across multiple days (17, 23, 27; see Section 3.1). Rapid learning is dominated by global strategies like reaiming (21). Longer timescales involve changes targeted to neurons used for BMI control (readouts) (17, 36, 39, 41). In contrast, learning in BMIs using the PPC for control appears to be dominated by rapid learning mechanisms. Both humans and animals struggle to learn PPC BMI mappings that cannot be solved by reaiming strategies, and neural changes are only global (19, 20). Differences in observed learning dynamics may stem from differences in computations performed by each area. The PPC is thought to represent primarily movement goals, which may constrain the types of learning computations it can perform to manipulations of goals. However, differences in BMI control used in each area (discrete control in PPC; continuous in M1) also influence the timing and forms of feedback available, which in turn may also influence learning (see Section 3.6).

Brain areas make distinct contributions to natural sensorimotor learning and control. Differences in how learning occurs across the motor system may influence closed-loop BMIs.

### 3.3. Neural Features

From the brain region(s) chosen to record from, BMIs compute neural features used for device control. These features are influenced by the sensors used and the signal processing applied to measurements. Recent reviews summarized differences between types of neural measurements and implications for decoding (62). In the following subsections, we highlight commonly used neural features and discuss how they may influence closed-loop BMI control and learning.

#### 3.3.1. Types of features and key differences.

Many BMIs use electrophysiologically measured action potentials. While early studies focused on well-isolated single neurons (25, 27), signals that combine action potentials from different neurons are now common,such as unsorted spiking activity (multiunit) data, threshold crossings (e.g., 1), and spike-band power (63). These features blend action potentials at the sensor level, grouping neurons that are physically nearby. An alternative is neural features that group neurons based on structure within neural populations. For example, correlations among action potentials have been used to define “latent factors”(18). These features blend action potentials based on statistical relationships independent of physical proximity. Latent factor decoding may be done with multiunit data, leading to spatial and statistical blending (64). Action potentials measured with calcium imaging have also been used for BMIs (39,65–68),which can provide cell type–specific (66) and subcellular (65) neural features.

The other main category of features is electrophysiological field potentials, which capture neural activity across a larger spatial area and on slower timescales than action potentials (69). The spatiotemporal properties of field potentials are influenced by sensor size, sensor placement (62), and signal frequency (low-frequency signals capture more global signals relative to higher frequencies). A wide variety of field potential measurements have been used in BMIs, including intracortical electrodes (LFPs) (52, 70–72) and electrodes on the cortical surface (ECoG) (28, 61, 73). Field potential features are most commonly defined in the spectral domain (e.g., power in a range of frequencies). BMIs often use features across a wide spectral range (e.g., 70), potentially blending neural activity across a variety of spatiotemporal scales.

Most closed-loop BMIs use a single feature type, but combinations of features have also been explored. A common combination is action potentials and LFPs, which can be recorded simultaneously from intracortical electrodes (52, 74).

#### 3.3.2. Engineering considerations.

Neural features differ in their temporal stability, affecting BMI usability and learning. Single-neuron signals are prone to drift with current electrophysiology technologies. Population-level features (46, 75) and field potentials (61, 71) are more stable, potentially through different mechanisms. Population-level features appear to capture computations performed by the population that remain stable even if the contributing neurons change (46). Field potentials are thought to capture similar neural activity over time as a result of physical properties of the measurements. Nonstationary neural features reduce decoding accuracy and require methods to adapt decoders (e.g., 75); however, frequent changes in neural features and decoders can reduce or eliminate learning (27, 51). Long-term ECoG BMI use leads to high performance and user learning (61, 73); population feature BMI studies have reported primarily maintenance of performance (75). Instability in features, or in the measured neural activity contributing to them, could affect learning processes such as credit assignment, reducing long-term learning. More research is needed to understand what forms of feature variability influence learning.

Neural features have different temporal resolutions, which affect closed-loop control and learning. The temporal resolution of a feature is tied to estimation methods. For instance, while action potentials are fast (millisecond resolution), most BMIs use the action potential rate, which is estimated by taking averages over time bins, constraining temporal resolution (76). This limitation can be overcome by using methods that directly estimate the underlying rate at millisecond resolution (76). The temporal resolution of features influences the rate of movement control and delay between neural activity and movement. In closed-loop systems, maximizing control rates (76) and minimizing delays (77) improve performance. Given the importance of sensorimotor timing for learning-related computations (78), timing differences in neural features may also affect learning, though this hypothesis remains to be explored.

The number of neural features used will influence both decoding performance and learning in BMIs. Open-loop decoding always benefits from adding information, but the relationship between the number of features and closed-loop performance is less well characterized and will depend on the features used. For instance, features that incorporate population dynamics can retain performance after losing many neurons due to redundancy (79). Learning is also influenced by the number of neurons used, though this effect has been explored only with small numbers (tens) of neurons (39, 80). How these findings extend to features often used in clinical BMIs is unclear. Using a large number of neural features may make it easier for the brain to find effective BMI solutions by increasing control redundancy (80), but how the number of features might influence learning processes like credit assignment is unknown.

#### 3.3.3. Biological considerations.

How neural features relate to the underlying functional neural circuitry must be considered. These considerations include the physical locations of neurons, cell types, and circuit connectivity—properties that are not completely independent. For instance, connectivity patterns differ across classes of inhibitory neurons (81).

Neural features differ in how they blend activity across neurons. Studies in which subjects control arbitrary BMI decoders suggest that increasing the physical distance between readout neurons negatively influences learning (80).How features that combine activity across large distances, such as population-based features, affect processes like credit assignment learning is unknown. A potentially related question concerns the impact of feature spatial resolution. BMI performance typically improves when features are spatially localized. For instance, action potentials provided better closed-loop performance than did locomotor potentials from LFPs, despite comparable offline predictive power (52).Whether these differences stem from the relative temporal or spatial resolution of these features has not been determined. Insights into the relevant scales of plasticity during motor learning will likely inform BMI feature design.

The relationship between features and anatomy may also influence learning. For instance, while cortical layers play distinct roles in natural motor learning (82), neural features used in BMIs often ignore layer boundaries, and layer-specific differences have been explored only in open-loop decoding (83). Interestingly, several studies have demonstrated distinct roles for cell types in BMIs. For example, bursting neurons adapt differently from nonbursting neurons during BMI learning (84).The cell types used for online BMI control also influence neural activity changes (66) and learning rates (85). Differences in both the anatomical and firing-rate properties between cell types contribute to these effects (85). How anatomical information can be leveraged to improve closed-loop BMIs remains an open question.

Neurons ultimately control movements through the activity of neuronal populations coordinated via anatomical and functional connections. Many neural features used in BMIs combine neural activity independent of anatomical connectivity. Features based on correlations in population activity, such as latent population dynamics, may provide one way to create features that reflect functional relationships.Decoder perturbations that do not require changes in population correlation structures are more rapidly learned than those that do not (18, 23).This finding highlights the potential importance of designing BMI features that capture functionally relevant circuit properties. Answering this question will require additional insight into how neural populations perform motor control and learning computations.

### 3.4. Decoders

The decoder defines how neural features map to movement. Properties of the decoder and how it is trained will influence closed-loop performance. We first review types of decoders and training methods used in closed-loop BMIs that will be relevant for this and subsequent sections. We then discuss how decoder training may influence learning.

#### 3.4.1. Decoder types and training methods.

Decoders can predict discrete or continuous movement variables. Discrete decoders perform classification to match a pattern of neural features to one of a finite list of possible movements. Discrete decoders often use neural features across a time interval longer than the time needed to estimate features, causing delays between neural activity and movement outcome. Continuous decoders map neural features to a continuously valued movement variable such as position or velocity. These decoders generally update movement at the same rate at which neural features are estimated.

The most common algorithms used in closed-loop BMIs linearly map neural features to movement. Directly mapping features to position is common in studies exploring learning of BMIs (25, 33, 36, 39, 68, 80). Algorithms like the Wiener filter (45) or the population vector algorithm and closely related variants (12) also directly map neural activity to movement variables. Alternately, decoders may map neural activity to a modeled state variable. The Kalman filter, for instance, predicts movements by combining a linear model of how the decoder state (e.g., velocity) relates to neural features with a model describing how the state evolves in time (86). Incorporating state models captures temporal dynamics, which play a role in closed-loop control (see Section 3.5). Nonlinear algorithms such as recurrent neural networks have also been used for both continuous (87, 88) and discrete (2) control.

Fully specifying a decoder requires setting its parameters. Parameters are often fitted to maximize the prediction of movement parameters from neural features on a training data set. Because predictive power on open-loop data is not predictive of closed-loop performance (12, 13), it is increasingly common for decoders to be trained on data collected as a user controls a closed-loop BMI. Methods to fully train or update decoder parameters in closed-loop BMIs can significantly improve performance (50, 89–91).

#### 3.4.2. Influence on performance and learning.

The timescales of decoder training influence closed-loop performance and user learning. A typical protocol involves the use of a finite period of training data to set parameters daily. This can be done without maintaining continuity in parameters across days (45, 50) or while accounting for measurement drift to maintain consistent feature–movement relationships (75). Alternatively, closed-loop decoder adaptation can update decoder parameters at some rate or frequency (51, 61, 92). Importantly,neural plasticity may occur alongside decoder changes, creating a coadaptive system (51, 89). Consequently, fully retraining decoders daily (including changes in neural features) disrupts learning (27, 51). In contrast, occasional decoder adaptation yields signatures of skill similar to those of fixed decoders, even with gradual drift in features (51, 61). Models have shown that coadaptive systems can become unstable if the algorithm and user learning rates are not appropriately matched (93). Understanding how decoder adaptation timescales influence performance and learning will be critical to optimize BMIs.

Adaptive decoders may also influence learning mechanisms, which have been studied primarily with fixed decoders. For example, learning a novel fixed decoder leads to significant changes in neural feature–movement relationships that gradually consolidate into a stable map (27). Periodic adaptive decoding also leads to a stable map, but reduces the amount of change in feature– movement relationships (51). In coadaptive BMIs, some of the largest learning-related changes are which neural features contribute to movement, rather than how they drive movement (51, 61). In light of the learning problems required for BMIs (see Section 3.1), this result demonstrates that adaptive decoding directly addresses only how neural features relate to movement. Whether and how adaptive decoding methods influence other key learning computations such as credit assignment remain to be fully explored.

### 3.5. Device and Control-Loop Properties

BMIs have controlled many devices, including virtual cursors (1, 45, 50, 51, 61) and robotic limbs (3, 4, 45). How the device moves in response to a given input—its dynamics—influences closed-loop BMI control. Changing the device being controlled from a cursor to a robot without changing the decoder leads to changes in performance (45). Device dynamics can also alter the timing of control (e.g., rates, delays), which affects performance (76, 77).

A BMI’s dynamics are governed not only by the physical properties of a device but also by the decoding algorithm. For instance, the Kalman filter models how movement states (e.g., position, velocity) evolve in time, thus describing system dynamics. The choice of movement states controlled by neural activity affects closed-loop performance. Simulators of closed-loop BMIs have shown that velocity-based control is more robust to input noise (94) because integration of velocity commands effectively denoises inputs. Importantly, the control variable defines the computations the brain must perform for learning and control.

The optimal device dynamics for BMI will be influenced by learning. In online control, brain learning can overcome performance differences due to changes in device dynamics (45). Such learning may employ similar mechanisms as natural motor adaptation to changing environmental dynamics, which is thought to rely on updates to an internal model of how the limb moves in response to inputs (5, 9, 24). Such predictive models are needed for feedforward control of movements. Evidence suggests that the brain builds internal models of a BMI device (95) and uses feedforward control strategies (76). Consistent with these findings, BMIs that constrain system dynamics to better match those of physical objects we regularly interact with significantly improve closed-loop performance, even when these constraints sacrifice open-loop prediction accuracy (50, 96, 97). This observation may shed light on why nonlinear algorithms have, to date, produced relatively modest performance improvements in closed-loop settings in comparison to their clear superiority to linear methods in open-loop prediction (88). Together, these results suggest the need for techniques to simultaneously optimize decoding accuracy and closed-loop device dynamics. Doing so will require new insights into how device parameters influence the ability to control and learn closed-loop BMI systems.

### 3.6. Forms of Feedback

Feedback closes the sensorimotor loop. This feedback can take many forms, including visual, tactile, auditory, and artificial (see Section 4). In BMIs, some form of feedback is necessary for goal-directed movement, real-time feedback control, and learning (25, 36, 76). The importance of feedback motivates research to provide artificial sensation, which is covered in the next section. Here, we briefly discuss how feedback influences motor control and learning.

The timing of feedback relative to actions is critical for sensorimotor control and learning. In the natural motor system, learned internal models are thought to allow the brain to anticipate the sensory consequences of motor commands and attenuate them to amplify errors. Disrupting the timing between movement and sensory consequences reduces this attenuation (98), thus influencing learning computations. While temporal delays occur in all BMIs, discrete BMIs typically introduce significant delays between neural activity and motor outcomes, which may contribute to differences in forms of learning observed in discrete versus continuous BMIs (e.g., compare 19 versus 41). The relative timing between movements and sensory feedback must be considered when optimizing BMIs.

The form of feedback must also be considered. Natural motor learning is driven by multiple types of error feedback, including sensory prediction errors (expected versus actual sensory consequences), reward prediction errors (expected versus actual outcomes), and task error (actual movement versus the goal). Each error signal contributes to different aspects of learning (9). Discrete feedback of movement outcomes, the only feedback available in discrete BMIs, largely eliminates sensory prediction error signals, while continuous BMIs provide all forms of feedback. The richer feedback signals available in continuous BMIs may contribute to differences in learning between the two. Indeed, BMI tasks that can be learned with continuous feedback cannot be learned with discrete task error feedback alone (success or failure) (36). Artificial stimulation of brain areas associated with reward can facilitate learning (33, 99), highlighting the contributions of reward prediction errors. Deeper insights into how sensory feedback signals contribute to control and learning in BMIs will place important constraints on the design of both motor and sensory devices.

## 4. ARTIFICIAL SENSATION FOR NEURAL PROSTHESES

### 4.1. Artificial Sensation

The goal of artificial sensation is to replace natural sensory information when sensory feedback circuits are interrupted by injury or disease. In a bidirectional BMI, artificial sensation would take the form of somatosensation: providing touch and proprioception that improve BMI control (100). People lacking one or the other sense are unable to manipulate small objects, maintain grip, or complete precise multijoint movements (101–103). BMI subjects using an artificial sense of touch have already gained agility in handling delicate objects (104); however, an artificial sense of proprioception remains elusive, despite its essential role during movement.

Artificial somatosensation can be provided to patients by modulating neural activity within sensorimotor circuits. To accomplish this goal, the scientific community must decide (*a*) how to modulate neural activity; (*b*) where to target neural modulation (anatomically); (*c*) how to encode touch and proprioception; and (*d*) what, if any, learning is required. Answers will depend on individual patients, the extent of their injuries, the physical demands of important behavioral tasks, and the type of neural prostheses they control. First, we restrict the problem space under discussion by considering only forms of neural modulation that can be directly translated into human patients (i.e., excluding optical techniques). Second, we focus on patients who have massive, widespread paralysis, which shifts the anatomical locus of the problem to the central nervous system. Finally, because we wish to describe the basic biological circuits involved in a sensory BMI, we consider only invasive neural modulation and its interaction with neural circuits.To satisfy these conditions, we discuss the use of electrical stimulation for artificial sensation.

An enduring technique for neural modulation is electrical stimulation, which consists of passing small electrical currents through an electrode. First implemented in the search for functional specialization across the nervous system in the nineteenth century, electrical stimulation facilitated the discovery of the sensory and motor homunculus—the idea that neurons within the sensory and motor areas of the brain are functionally organized according to the part of the body to which they respond (105). Since then, electrical stimulation has formed the basis of experiments causally linking neural activity within specific brain regions to complex behavior and cognition (106, 107). In our modern era, electrical stimulation is additionally employed in the pursuit of artificial sensation, finding clinical success in cochlear implants (108) and enabling progress in the development of artificial somatosensation.

### 4.2. Brain Regions to Target for Artificial Feedback

The pathway traveled by touch and proprioceptive information during natural sensation has been well described (102), but we summarize it here briefly in order to gain insight into which brain areas should be targeted for artificial somatosensation. Sensory processing is organized hierarchically: Signals from sensory receptors on the periphery are sent to the spinal cord and onward into the brainstem, thalamus, and primary somatosensory cortex. Touch and proprioceptive information split in the thalamus, from which information about touch is sent primarily to areas 3b and 1 within the somatosensory cortex, while information about proprioception is sent primarily to areas 3a and 2. From the somatosensory cortex, information flow splits into two parallel streams whose functions diverge:

1. Higher-level feature extraction occurs along the ventral stream, including the secondary somatosensory cortex and parietal ventral area.
2. Motor planning occurs along the dorsal stream, which includes areas 5 and 7.

Neural responses within each brain area can be characterized by their stimulus tuning (responses to different parameters of a stimulus) and receptive fields (areas of the body to which a neuron is responsive). Receptive field sizes grow along the sensory processing hierarchy, as information originating from multiple sensors is integrated to implement higher-level functions such as object recognition or movement planning. The activity of a neuron within the somatosensory stream can be thought of as a “vote” for the type of sensory information to which it is tuned. Therefore, activation of a neuron via electrical stimulation can be used to tell the brain that a particular type of sensory information is present.

The location of stimulation, including the type of neurons that are stimulated and their relation to the behavioral task of interest, seems to be critical for effective stimulation, yet there are only minor differences in animals’ ability to detect stimulation across different brain regions (109, 110). Despite this apparent flexibility, there are several reasons that most studies have focused on the early somatosensory cortex (111–115). First, its superficial position upon the cortical surface makes the somatosensory cortex more accessible than the thalamus or spinal cord. Second, neurons within the somatosensory cortex tend to have smaller receptive fields than higher-level areas, which means that smaller portions of the body can be targeted for selective stimulation. Third, more neurons are devoted to each body part in the somatosensory cortex than in earlier parts of the nervous system, making it easy to target many locations to encode complex information. A motivating example is the hand, for which high-dimensional feedback will be needed to control grasp and detect touch in the fingers.

An argument can still be made for targeting higher cortical areas. For instance, the receptive fields of neurons within area 5 are larger than in the somatosensory cortex, often spanning multiple joints, and are mostly proprioceptive in nature. Therefore, it is possible that stimulation at a single site in area 5 would encode a set of joint angles for the entire upper limb—something that would require multichannel stimulation within the somatosensory cortex. However, if electrical stimulation evokes unusual neural activity patterns within the brain, stimulation of higher cortical areas may be more likely to disrupt sensorimotor function than would stimulation of early somatosensory regions. Therefore, although multiple brain regions could serve as viable targets for artificial sensation, there are many reasons to assert that the primary somatosensory cortex is an ideal choice.

### 4.3. Neural Activity Patterns Evoked by Electrical Stimulation

**Figure 3:**
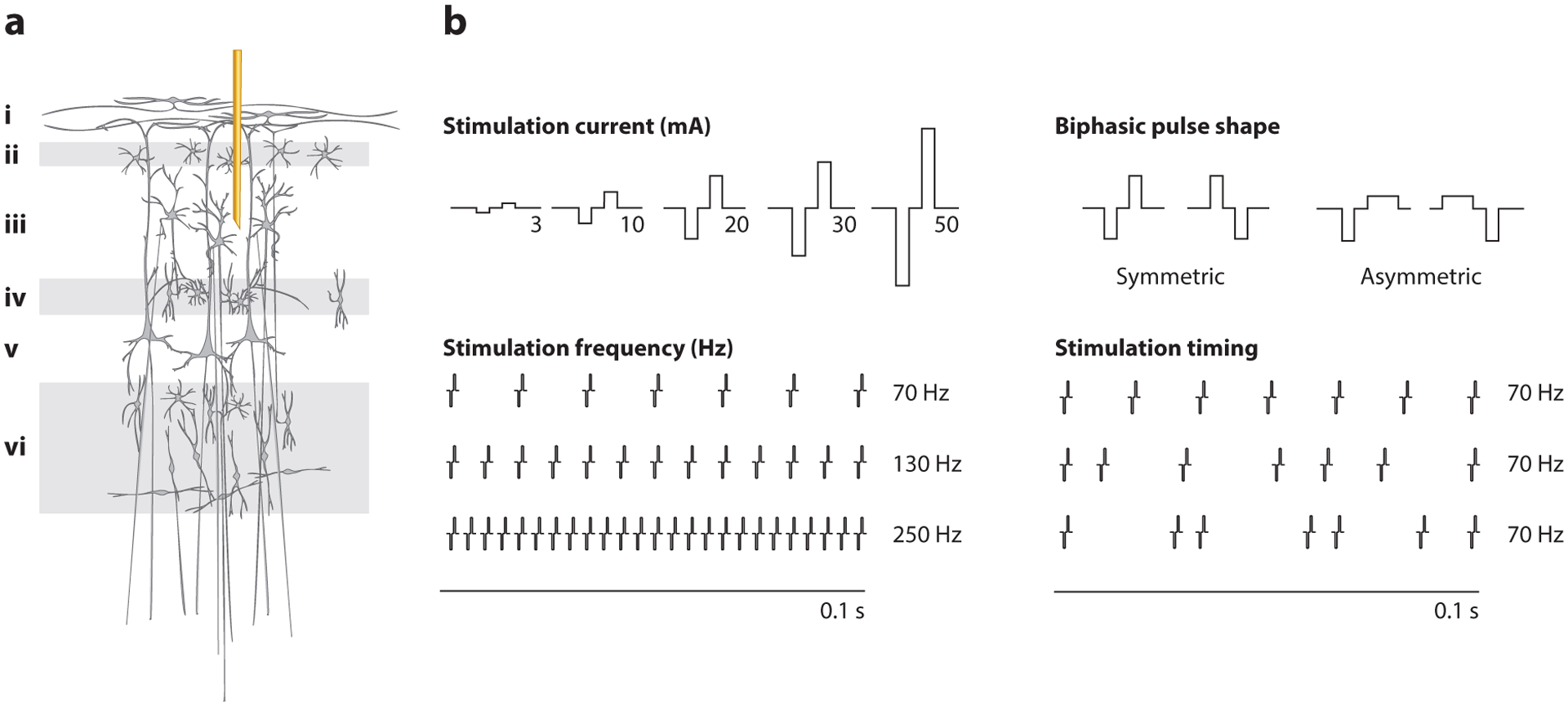
(*a*) A stimulating microelectrode is implanted in layer *iii* of the cortex. (*b*) Parameters that can be manipulated during electrical stimulation of brain tissue include the amplitude, frequency, timing, and shape of biphasic pulses.

The brain represents natural sensory information in the number and timing of neural action potentials. Therefore, to design effective artificial sensation, we must understand how the spatial and temporal pattern of stimulation-evoked neural responses depend on the parameters of electrical stimulation. The smallest unit of electrical stimulation is a constant-current biphasic pulse, which minimizes damage to both brain and electrode. Stimulation often consists of a train of pulses, which can vary in timing (stimulation frequency and pulse timing), stimulation amplitude, and the shape of the biphasic waveform (Figure 3). Each of these parameters affects stimulation-evoked neural activity patterns.

Traditionally, a single pulse of electrical stimulation was thought to activate neurons within a sphere surrounding the electrode tip (116). An increase in the stimulation amplitude increased the number of activated neurons and extended their physical spread (117). However, more recent research found that low-amplitude electrical stimulation activated only a sparse, distributed set of neurons whose axons were proximal to the electrode tip (118), and an increase in stimulation amplitude seemed to fill in the space by recruiting more neurons within the same space. A possible resolution has been proposed, asserting that while stimulation does activate axons that pass by the electrode tip, the likelihood of directly activating a cell body depends on the distance between the neuron and the electrode tip (119).

Stimulation frequency, pulse timing, and pulse shape can also control stimulation-evoked neural activity patterns (Figure 3). Stimulation frequency affects the spatial extent of stimulation-evoked activity (120), the population of neurons that are recruited, and the type of responses that are elicited (inhibitory versus excitatory) (121). The specific temporal pattern of pulse delivery also matters, such that small differences in timing change the population of recruited neurons (122). Finally, the application of asymmetric waveforms reduces the spatial extent of neural activation by electrical stimulation (123). Ongoing animal behavior at the time of stimulation also affects evoked neural activity; stimulation delivered during movement is less effective at activating neural responses (124) and can actively suppress neural activity (125). However, the interaction between behavior and stimulation-evoked neural activity has yet to be explored in depth, despite its clear importance.

Stimulation-evoked neural activity patterns can be made to resemble neural activity during natural sensory processing by careful manipulation of stimulation parameters. In one study, for example, controlling the timing and amplitude of a train of stimulation pulses in a pattern designed using a recurrent neural network can make stimulation-evoked spiking patterns look more natural (126). The only limitation of this study is that the algorithm was optimized to manipulate the activity a single neuron without considering the effects on surrounding neurons, thus ignoring spatial activation patterns. However, the study presents a compelling proof of concept in combination with published literature demonstrating spatial control over neural activity patterns via manipulation of natural stimuli (127). Future research will need to focus on higher-dimensional control over stimulation-evoked neural activity.

### 4.4. Learning to Use Artificial Sensation

The goal of artificial somatosensation is to enable naturalistic control of an external device in a bidirectional BMI. However, stimulation-evoked neural activity generally does not look natural in the spatial patterns of activation (118). Therefore, we have a critical open question to address: Does artificial sensation have to be learned?

#### 4.4.1. Direct transfer of performance.

Animal behavioral experiments show that electrical stimulation can directly replace natural sensation, particularly the sense of touch. Monkeys trained to compare the frequencies of mechanical vibrations were equally able to compare the frequencies of mechanical and electrical stimuli without any further training (128) if certain stimulation parameters were used. Similarly, monkeys trained to detect and discriminate mechanical touch to different fingers on the hand can, without training, complete the task when electrical stimulation of area 3b or area 1 replaces one or both natural stimuli (112). Rodents, too, can substitute electrical stimulation with natural touch, but only if stimulation is targeted to the barrel cortex rather than the trunk or hindlimb areas (129).

Examples of direct transfer extend beyond the somatosensory cortex. Rats trained to perform a tone discrimination task could immediately transfer performance when the sounds were replaced by electrical stimulation (130), and could generalize task performance to new stimulation sites without further training (131). Therefore, although direct transfer between natural sensation and electrical stimulation is possible, the location and stimulation parameters must be carefully chosen.

#### 4.4.2. Learning-based artificial sensation.

In contrast to the examples of direct transfer discussed in the preceding section, most behavioral studies involving artificial sensation require some training, where an animal gradually learns to detect or discriminate patterns of electrical stimulation. Such learning-based approaches can be divided into passive and active sensing. During passive sensing, animals receive stimulation as a cue to complete some action, such as reaching or gazing toward a target. Researchers then change the stimulation patterns in order to query perception and stimulus discriminability (e.g., 115, 132). Active sensing, on the other hand, more closely resembles natural sensorimotor function, where the timing and parameters of stimulation change as a function of the animal’s behavior.

Passive sensing has provided a rich body of data on how well animals can distinguish between electrical stimulation with different parameters, and how those parameters can be mapped back to natural sensation. Animals detect stimuli as weak as a single pulse of electrical stimulation (133), even at amplitudes that are close to the threshold for evoking neural activity (117), and remain sensitive to stimulation for years (134); however, increasing stimulation amplitude makes artificial sensation easier to detect (133). Animals can distinguish the temporal pattern of stimulation within the primary somatosensory cortex, the electrode across which stimulation is being delivered (113, 135), the frequency of stimulation (132), and even the precise timing of stimulation pulses (136). Although amplitude and frequency can be controlled independently during stimulation, these two parameters are not always perceptually dissociable (132). Note that these behavioral results describing the discriminability of stimulation parameters mirror the differences in stimulation-evoked neural activity patterns described above, identifying stimulation pulse timing, frequency, amplitude, and location as parameters that can be manipulated to elicit distinct sensations.

Active sensing studies serve as a proof of concept that electrical stimulation can provide interactive, real-time information to represent internal and external stimuli. For example, electrical stimulation can transform inaccessible sensory information in the environment into something that we can understand and use. Rats, like most rodents, are normally unable to detect infrared (IR) light; however, by translating measurements from a head-mounted IR sensor into the frequency of cortical electrical stimulation, they can localize IR-emitting targets within their environment (137). Similarly, rats can use stimulation signals to guide their progress through a Morris water maze, where stimulation provides information about target location (138). Both of these results are possible only if the animals integrated stimulation timing with information about their own position and heading in space.

Another compelling example of active integration is a study where animals had to determine which of two “virtual textures” had a higher spatial frequency (114). In this task, monkeys received an electrical stimulation pulse whenever their fingers passed over a virtual ridge, so the timing and frequency of electrical stimulation depended on the movements of the animal itself as well as on the spatial frequency of the virtual texture. To properly discriminate between the two, the animal needed to integrate stimulus timing with the location and speed of its hand moving across the workspace. Therefore, for animals to complete this and prior behavioral tasks, artificial sensation had to be integrated into the normal sensorimotor processing loop in order to help the animals plan their movements.

In addition to sensorimotor integration, a critical aspect of natural sensation is integration with other forms of sensory information. For example, during natural reaching, humans make use of both proprioceptive and visual information to plan and guide their movements (139) in a manner that depends on the relative reliability of the inputs (140). Experiments have found that electrical stimulation can indeed be integrated with both other sensory cues and motor plans. Monkeys trained to use visual flow fields or multichannel stimulation to reach to invisible targets ultimately integrate the two signals “optimally” on the basis of their relative reliability (111, 141). Human patients also integrate artificial sensation optimally with natural vision in order to estimate the size of a handheld object (142). Thus, artificial sensory information can be integrated with natural sensory streams reporting information about both the external environment and the internal state of the user.

In the active sensing experiments described above, animals learned to integrate natural sensorimotor information with the timing and location of stimulation, even though patterns of electrical stimulation did not imitate local neural activity patterns and instead had to be learned. These results suggest that learning-based stimulation is sufficient for artificial sensation; however, the behavioral tasks described were relatively simple, so the information needed was relatively low dimensional. In contrast, control over a prosthetic arm will require higher-dimensional information, including the position and rotational speed of each joint as well as touch across the surface of the limb. Therefore, as the complexity of the problem scales, we may have to be more careful about the learning load imposed on the user, but for simpler tasks, a learning-based approach is highly effective.

### 4.5. Cortical Adaptation to Electrical Stimulation

Any act of learning requires neural plasticity, defined as the ability of neurons to change the strength and pattern of their connections as a result of experience. Natural sensorimotor learning involves plasticity within the basal ganglia and the sensory, motor, and prefrontal cortices—brain areas that are responsible for adapting to new sensory information and associating sensory inputs with motor commands (143). Learning in a motor BMI also involves plasticity in, at a minimum, the basal ganglia and motor cortex (144), while learning to detect electrical stimulation requires (at least) plasticity in sensory cortical areas (145). However, electrical stimulation drives cortical plasticity even in the absence of any behavioral relevance (146, 147), so learning within the context of a BMI will consist of a balance between adapting to stimulation-evoked responses, assigning stimulation-evoked responses behavioral relevance, and associating stimulation-evoked responses with motor commands.

#### 4.5.1. Stimulation-evoked neuroplasticity in the absence of overt behavior.

Neurons undergo both homeostatic plasticity, which maintains steady activity levels over time, and Hebbian plasticity, a form of activity-dependent plasticity (148). Scientists have successfully employed Hebbian plasticity to strengthen specific connections within the cortex, by pairing neural activity on a recording electrode with electrical stimulation on a second electrode. Neural plasticity can be induced quickly this way, within 48 h of stimulation (149–151), and the effects persist for hours. However, timing is important: Plasticity is induced only with low-latency stimulation (5–50 ms after an action potential) (149, 150).

Increasing the functional connectivity between neurons has significant behavioral consequences. Pairing stimulation across two electrodes lowers the threshold for stimulus detection on a single electrode (151), making the stimulus more salient. Furthermore, spike-triggered stimulation within the motor cortex reorganizes motor output, shifting the muscle activated by the recorded neuron toward that of the stimulated one (presumably by strengthening the lateral connections between neurons) (149). Paired stimulation has clinical applications, such as forging new connections to circumvent brain areas damaged by stroke or disease.

Plasticity in neural responses can also be induced by electrical stimulation in the absence of specific pairings. For example, cortical electrical stimulation correlates spontaneous spiking in a population of neurons (147) and shifts neural receptive fields toward those at the stimulation site, expanding the total cortical area dedicated to a single part of the body (146). Nearly identical shifts follow prolonged exposure to natural stimuli (152), indicating shared mechanisms of plasticity between natural and artificial sensation.

#### 4.5.2. Learning to detect electrical stimulation.

Adding behavioral relevance to electrical stimulation is at the heart of artificial sensation; however, surprisingly little is known about how neurons adapt their responses to behaviorally significant stimulation. Single neurons do seem to change their responses in subtle ways. Neurons typically respond to electrical stimulation by a short burst of excitation followed by a longer-lasting inhibition (117). After behavioral training, neurons have both a larger active response and a longer inhibitory phase compared with baseline (145). These changes show clear adaptation to novel experience.

Additional evidence reveals circuit-level adaptation during learning to detect electrical stimulation. Nonhuman primates can learn to detect stimulation at amplitudes as low as 5 μA (153), at which only a sparse set of neurons is activated (118). The lower bound on conscious detection of neural activity is thought to be around 14 neurons within the upper layers of the mouse somatosensory cortex (154), although further training can lower this bound to a single neuron (155). Neural prostheses will ultimately require a large number of stimulating channels to encode high-dimensional sensory information, which will be easier to implement if only a small number of neurons are needed for detection on each channel.

Behavioral experiments that involve learning to detect electrical stimulation have provided some insight into how neural circuits might adapt during learning. Animals can detect the presence of weak natural and electrical stimuli with practice; however, learning to detect electrical stimulation seems to compete with detection of natural stimuli (153). Therefore, learning to detect electrical stimulation seems to require plasticity to optimize neural circuits for a particular stimulation-evoked neural activity pattern. In contrast, training mice to control a simple BMI does not interfere with responses to natural stimuli (156). Thus, it is possible that different mechanisms of learning and plasticity are employed for sensory perception than for de novo motor learning, even within the sensory cortices.

### 4.6. Sensory Percepts Elicited by Electrical Stimulation

Electrical stimulation can either elicit overt sensations or simply bias the perception of natural stimuli (157). Animal models provide only indirect evidence regarding stimulation-evoked sensations, with mixed results suggesting both naturalistic sensations (112, 128, 130, 131, 158) and unnatural sensations (111, 114, 135, 136). Humans, on the other hand, can simply tell us how electrical stimulation feels.

Electrical stimulation in humans that was used to map cortical function in the course of surgery induced sensations such as numbness, tingling, and “as though it was going to sleep” (105). Only in recent research with penetrating microelectrodes have stimulation-evoked sensations come to have a more “natural” feel, including a mix of natural (mechanical, movement, temperature) and unnatural (tingling, vibration) sensations (115, 159). In addition to the location and type of electrodes used, the parameters chosen for electrical stimulation affect evoked sensations. Humans perceive the intensity of a stimulation-induced sensation to increase linearly with stimulation amplitude and duration (115). In contrast, increasing stimulation frequency has mixed effects on perception,at some times heightening and at others weakening the perceived intensity of the stimulus (159). Note that most of the sensations described above have qualities of touch; proprioceptive-like sensations were found at higher stimulation amplitudes and frequencies (160). What has yet to be fully explored in humans is how precise timing affects evoked sensations (but see 161).

Electrical stimulation seems to elicit mixed sensations: some that feel natural and others that do not. However, we argue that sensation need not feel natural to be useful during behavioral tasks. Humans were able to use peripheral nerve stimulation as part of a bidirectional BMI, even when the sensations elicited by stimulation felt unnatural (162). Furthermore, as discussed above, animals learned to integrate electrical stimulation cues into natural sensory and motor behavior, even where stimulation patterns did not imitate natural neural activity patterns. From these studies, we conclude that stimulation-evoked sensations that convey graded information to the nervous system are already accomplishing the ultimate goal of artificial sensation in a sensory or bidirectional BMI—enabling accurate, precise sensorimotor function. We go so far as to speculate that stimulation will, over time, start to feel natural as it acquires higher-level meaning during closed-loop behavior.

## 5. CONCLUSIONS AND FUTURE RESEARCH

In this review, we have discussed sensory and motor BMIs separately, because the complexities of each pathway have resulted in largely distinct lines of research focused on optimizing each component in isolation. However, implementing bidirectional BMI experiments will be critical to achieving high levels of performance for both motor readout and sensory processing. Even when considering motor or sensory BMIs separately, research has revealed the importance of closed-loop sensorimotor interactions and the need for new engineering approaches to optimize closed-loop BMIs.

While feedback like vision allows almost all motor BMIs to be closed-loop systems, movement precision and real-world interactions will be limited in the absence of proprioceptive and tactile feedback (4, 103). Advances in motor BMIs require that we move past purely open-loop machine learning approaches to optimize closed-loop performance. Doing so will require careful consideration of all aspects of the system, from neural features to control dynamics, and how they interact with the brain’s learning computations.

From the sensory side, future experiments must be closed-loop to promote learning and plasticity. Although we have learned much about how electrical stimulation is processed using passive sensing alone, the most complex behavioral tasks achieved with electrical stimulation were closed-loop, where stimulation was delivered as a function of the animal’s behavior rather than passively received prior to behavior. In imitation of natural sensorimotor processing, we must train BMIs by taking advantage of three levels of neural plasticity: adaptation to motor control algorithms, adaptation to electrical stimulation inputs, and a learned mapping between the two.

Much as natural sensory and motor functions codevelop, optimizing bidirectional BMIs will ultimately require joint design of artificial sensory and motor pathways. Such design will require insight into the principles of neural plasticity in sensorimotor BMIs, as well as new engineering methods for closed-loop optimization. The future of BMI must be bidirectional.

## SUMMARY POINTS

1. Sensory and motor neural circuits form through paired experience and continually adapt together to control sensorimotor function.
2. The inclusion of sensory feedback, whether natural or artificial, in motor brain–machine interfaces (BMIs) leads to learning and error-based corrections that in turn lead to performance differences relative to predicting motor intent alone.
3. All elements of a motor MBI together define a sensorimotor transformation that influences the neural computations for learning and control.
4. Artificial sensation is integrated with natural sensory and motor processing during closed-loop sensorimotor behaviors.
5. Neural circuits adapt to continued electrical stimulation, even in the absence of behavioral relevance.
6. The tight link between sensory inputs and motor outputs in closed-loop BMIs engages innate neural mechanisms for learning, thereby changing the nature of engineering problems that must be solved to optimize BMI performance.

## FUTURE ISSUES

1. Algorithms to train closed-loop BMIs must include constraints informed by learning and control mechanisms in the brain.
2. Design of optimal stimulation patterns for artificial sensation must include closed-loop experiments, where behavioral relevance will drive plasticity and learning.

## References

[1] Pandarinath C, Nuyujukian P, Blabe CH, Sorice BL, Saab J, 2017. High performance communication by people with paralysis using an intracortical brain-computer interface. eLife 6:e18554 28220753 10.7554/eLife.18554 PMC5319839

[2] Willett FR, Avansino DT, Hochberg LR, Henderson JM, Shenoy KV. 2022. High-performance brain-to-text communication via handwriting. Nature 593(7858):249–54 10.1038/s41586-021-03506-2 PMC8163299 33981047

[3] Collinger JL, Wodlinger B, Downey JE, Wang W, Tyler-Kabara EC, 2013. High-performance neuroprosthetic control by an individual with tetraplegia. Lancet 381(9866):557–64 23253623 10.1016/S0140-6736(12)61816-9 PMC3641862

[4] Flesher SN, Downey JE, Weiss JM, Hughes CL, Herrera AJ, 2021. A brain-computer interface that evokes tactile sensations improves robotic arm control. Science 372(6544):831–36 34016775 10.1126/science.abd0380 PMC8715714

[5] Wolpert D, Ghahramani Z, Jordan M. 1995. An internal model for sensorimotor integration. Science 269(5232):14–16 7569931 10.1126/science.7569931

[6] Hein A, Held R. 1967. Dissociation of the visual placing response into elicited and guided components. Science 158(3799):390–92 6061894 10.1126/science.158.3799.390

[7] Hein A, Held R, Gower EC. 1970. Development and segmentation of visually controlled movement by selective exposure during rearing. J. Comp. Physiol. Psychol 73(2):181–87 5493257 10.1037/h0030247

[8] Attinger A, Wang B, Keller GB.2017.Visuomotor coupling shapes the functional development of mouse visual cortex. Cell 169(7):1291–302.e14 28602353 10.1016/j.cell.2017.05.023

[9] Krakauer JW, Hadjiosif AM, Xu J, Wong AL, Haith AM.2019.Motor learning.Compr.Physiol 9(2):613–63 30873583 10.1002/cphy.c170043

[10] Bastian AJ. 2008. Understanding sensorimotor adaptation and learning for rehabilitation. Curr. Opin. Neurol 21(6):628–33 18989103 10.1097/WCO.0b013e328315a293 PMC2954436

[11] Henriques DYP, Cressman EK. 2012. Visuomotor adaptation and proprioceptive recalibration. J. Mot. Behav 44(6):435–44 23237466 10.1080/00222895.2012.659232

[12] Chase SM, Schwartz AB, Kass RE. 2009. Bias, optimal linear estimation, and the differences between open-loop simulation and closed-loop performance of spiking-based brain–computer interface algorithms. Neural Netw. 22(9):1203–13 19502004 10.1016/j.neunet.2009.05.005 PMC2783655

[13] Koyama S, Chase SM, Whitford AS, Velliste M, Schwartz AB, Kass RE. 2010. Comparison of brain– computer interface decoding algorithms in open-loop and closed-loop control. J. Comput. Neurosci 29(1):73–87 19904595 10.1007/s10827-009-0196-9

[14] Orsborn AL, Pesaran B. 2017. Parsing learning in networks using brain–machine interfaces. Curr. Opin. Neurobiol 46:76–83 28843838 10.1016/j.conb.2017.08.002 PMC5660637

[15] Jarosiewicz B, Chase SM, Fraser GW, Velliste M, Kass RE, Schwartz AB. 2008. Functional network reorganization during learning in a brain-computer interface paradigm. PNAS 105(49):19486–91 19047633 10.1073/pnas.0808113105 PMC2614787

[16] Chase SM, Kass RE, Schwartz AB. 2012. Behavioral and neural correlates of visuomotor adaptation observed through a brain-computer interface in primary motor cortex. J. Neurophysiol 108(2):624–44 22496532 10.1152/jn.00371.2011 PMC3404791

[17] Zhou X, Tien RN, Ravikumar S, Chase SM. 2019. Distinct types of neural reorganization during long-term learning. J. Neurophysiol 121(4):1329–41 30726164 10.1152/jn.00466.2018 PMC6485743

[18] Sadtler PT, Quick KM, Golub MD, Chase SM, Ryu SI, 2014. Neural constraints on learning. Nature 512(7515):423–26 25164754 10.1038/nature13665 PMC4393644

[19] Hwang E, Bailey P, Andersen R. 2013. Volitional control of neural activity relies on the natural motor repertoire. Curr. Biol 23(5):353–61 23416098 10.1016/j.cub.2013.01.027 PMC3633426

[20] Sakellaridi S, Christopoulos VN, Aflalo T, Pejsa KW, Rosario ER,.2019.Intrinsic variable learning for brain-machine interface control by human anterior intraparietal cortex. Neuron 102(3):694–705.e3 30853300 10.1016/j.neuron.2019.02.012 PMC6922088

[21] Golub MD, Sadtler PT, Oby ER, Quick KM, Ryu SI, 2018. Learning by neural reassociation. Nat. Neurosci 21(4):607–16 29531364 10.1038/s41593-018-0095-3 PMC5876156

[22] Taylor JA, Krakauer JW, Ivry RB.2014.Explicit and implicit contributions to learning in a sensorimotor adaptation task. J. Neurosci 34(8):3023–32 24553942 10.1523/JNEUROSCI.3619-13.2014 PMC3931506

[23] Oby ER, Golub MD, Hennig JA, Degenhart AD, Tyler-Kabara EC, 2019. New neural activity patterns emerge with long-term learning. PNAS 116(30):15210–15 31182595 10.1073/pnas.1820296116 PMC6660765

[24] Yang CS, Cowan NJ, Haith AM. 2021. De novo learning versus adaptation of continuous control in a manual tracking task. eLife 10:e62578 34169838 10.7554/eLife.62578 PMC8266385

[25] Fetz EE. 1969. Operant conditioning of cortical unit activity. Science 163(3870):955–58 4974291 10.1126/science.163.3870.955

[26] Moritz CT, Fetz EE. 2011. Volitional control of single cortical neurons in a brain–machine interface. J. Neural Eng 8:025017 21436531 10.1088/1741-2560/8/2/025017 PMC3156089

[27] Ganguly K, Carmena JM. 2009. Emergence of a stable cortical map for neuroprosthetic control. PLOS Biol. 7(7):e1000153 19621062 10.1371/journal.pbio.1000153 PMC2702684

[28] Wander JD, Blakely T, Miller KJ, Weaver KE, Johnson LA, 2013. Distributed cortical adaptation during learning of a brain–computer interface task. PNAS 110(26):10818–23 23754426 10.1073/pnas.1221127110 PMC3696802

[29] Gulati T, Ramanathan DS, Wong CC, Ganguly K. 2014. Reactivation of emergent task-related ensembles during slow-wave sleep after neuroprosthetic learning. Nat. Neurosci 17(8):1107–13 24997761 10.1038/nn.3759 PMC5568667

[30] Gulati T, Guo L, Ramanathan DS, Bodepudi A, Ganguly K. 2017. Neural reactivations during sleep determine network credit assignment. Nat. Neurosci 20(9):1277–84 28692062 10.1038/nn.4601 PMC5808917

[31] Dayan E, Cohen LG. 2011. Neuroplasticity subserving motor skill learning. Neuron 72(3):443–54 22078504 10.1016/j.neuron.2011.10.008 PMC3217208

[32] Athalye VR, Ganguly K, Costa RM, Carmena JM. 2017. Emergence of coordinated neural dynamics underlies neuroprosthetic learning and skillful control. Neuron 93(4):955–70.e5 28190641 10.1016/j.neuron.2017.01.016

[33] Athalye VR, Santos FJ, Carmena JM, Costa RM. 2018. Evidence for a neural law of effect. Science 359(6379):1024–29 29496877 10.1126/science.aao6058

[34] Peters AJ, Chen SX, Komiyama T. 2014. Emergence of reproducible spatiotemporal activity during motor learning. Nature 510(7504):263–67 24805237 10.1038/nature13235

[35] Dhawale AK, Smith MA, Ölveczky BP. 2017. The role of variability in motor learning. Annu. Rev. Neurosci 40:479–98 28489490 10.1146/annurev-neuro-072116-031548 PMC6091866

[36] Koralek AC, Jin X, Long JD II, Costa RM, Carmena JM. 2012. Corticostriatal plasticity is necessary for learning intentional neuroprosthetic skills. Nature 483(7389):331–35 22388818 10.1038/nature10845 PMC3477868

[37] Koralek A, Costa R, Carmena J. 2013. Temporally precise cell-specific coherence develops in corticostriatal networks during learning. Neuron 79(5):865–72 23954030 10.1016/j.neuron.2013.06.047

[38] Liu Z, Schieber MH. 2020. Neuronal activity distributed in multiple cortical areas during voluntary control of the native arm or a brain-computer interface. eNeuro 7(5):ENEURO. 0376–20.2020 10.1523/ENEURO.0376-20.2020 PMC7598906 33060178

[39] Clancy KB, Koralek AC, Costa RM, Feldman DE, Carmena JM.2014.Volitional modulation of optically recorded calcium signals during neuroprosthetic learning. Nat. Neurosci 17(6):807–9 24728268 10.1038/nn.3712 PMC4361947

[40] Arduin PJ, Fregnac Y, Shulz DE, Ego-Stengel V. 2013. “Master” neurons induced by operant conditioning in rat motor cortex during a brain-machine interface task. J. Neurosci 33(19):8308–20 23658171 10.1523/JNEUROSCI.2744-12.2013 PMC6619624

[41] Ganguly K, Dimitrov DF, Wallis JD, Carmena JM. 2011. Reversible large-scale modification of cortical networks during neuroprosthetic control. Nat. Neurosci 14(5):662–67 21499255 10.1038/nn.2797 PMC3389499

[42] Gallego JA, Makin TR, McDougle SD. 2022. Going beyond primary motor cortex to improve braincomputer interfaces. Trends Neurosci. 45(3):176–83 35078639 10.1016/j.tins.2021.12.006

[43] Dum R, Strick PL.2004.Motor areas in the frontal lobe: the anatomical substrate for the central control of movement. In Motor Cortex in Voluntary Movements: A Distributed System for Distributed Functions, ed. Riehle A, Vaadia E, pp. 3–47. Boca Raton, FL: CRC

[44] Omrani M, Kaufman MT, Hatsopoulos NG, Cheney PD. 2017. Perspectives on classical controversies about the motor cortex. J. Neurophysiol 118(3):1828–48 28615340 10.1152/jn.00795.2016 PMC5599665

[45] Carmena JM, Lebedev MA, Crist RE, O’Doherty JE, Santucci DM, 2003. Learning to control a brain–machine interface for reaching and grasping by primates. PLOS Biol. 1(2):e42 14624244 10.1371/journal.pbio.0000042 PMC261882

[46] Gallego JA, Perich MG, Chowdhury RH, Solla SA, Miller LE. 2020. Long-term stability of cortical population dynamics underlying consistent behavior. Nat. Neurosci 23(2):260–70 31907438 10.1038/s41593-019-0555-4 PMC7007364

[47] Cherian A, Krucoff MO, Miller LE. 2011. Motor cortical prediction of EMG: evidence that a kinetic brain-machine interface may be robust across altered movement dynamics. J. Neurophysiol 106(2):564–75 21562185 10.1152/jn.00553.2010 PMC3154818

[48] Cisek P, Kalaska JF.2005. Neural correlates of reaching decisions in dorsal premotor cortex: specification of multiple direction choices and final selection of action. Neuron 45(5):801–14 15748854 10.1016/j.neuron.2005.01.027

[49] Shanechi MM, Hu RC, Powers M, Wornell GW, Brown EN, Williams ZM. 2012. Neural population partitioning and a concurrent brain-machine interface for sequential motor function. Nat. Neurosci 15(12):1715–22 23143511 10.1038/nn.3250 PMC3509235

[50] Gilja V, Nuyujukian P, Chestek CA, Cunningham JP, Yu BM, 2012. A high-performance neural prosthesis enabled by control algorithm design. Nat. Neurosci 15(12):1752–57 23160043 10.1038/nn.3265 PMC3638087

[51] Orsborn AL, Moorman H, Overduin S, Shanechi M, Dimitrov D, Carmena JM. 2014. Closed-loop decoder adaptation shapes neural plasticity for skillful neuroprosthetic control. Neuron 82(6):1380–93 24945777 10.1016/j.neuron.2014.04.048

[52] Stavisky SD, Kao JC, Nuyujukian P, Ryu SI, Shenoy KV. 2015. A high performing brain–machine interface driven by low-frequency local field potentials alone and together with spikes. J. Neural Eng 12:036009 25946198 10.1088/1741-2560/12/3/036009 PMC4457459

[53] Mulliken GH, Musallam S, Andersen RA. 2008. Decoding trajectories from posterior parietal cortex ensembles. J. Neurosci 28(48):12913–26 19036985 10.1523/JNEUROSCI.1463-08.2008 PMC2728059

[54] Whitlock JR. 2017. Posterior parietal cortex. Curr. Biol 27(14):R691–95 28743011 10.1016/j.cub.2017.06.007

[55] Andersen RA, Buneo CA. 2002. Intentional maps in posterior parietal cortex. Annu. Rev. Neurosci 25:189–220 12052908 10.1146/annurev.neuro.25.112701.142922

[56] Batista AP, Buneo CA, Snyder LH, Andersen RA.2022.Reach plans in eye-centered coordinates.Science 285(5425):257–60 10.1126/science.285.5425.257 10398603

[57] Neely RM, Koralek AC, Athalye VR, Costa RM, Carmena JM. 2018. Volitional modulation of primary visual cortex activity requires the basal ganglia. Neuron 97(6):1356–68.e4 29503189 10.1016/j.neuron.2018.01.051

[58] Musall S, Kaufman MT, Juavinett AL, Gluf S, Churchland AK. 2019. Single-trial neural dynamics are dominated by richly varied movements. Nat. Neurosci 22(10):1677–86 31551604 10.1038/s41593-019-0502-4 PMC6768091

[59] Ganguly K, Secundo L, Ranade G, Orsborn AL, Chang EF, 2009. Cortical representation of ipsilateral arm movements in monkey and man. J. Neurosci 29(41):12948–56 19828809 10.1523/JNEUROSCI.2471-09.2009 PMC3376707

[60] Mahmoudi B, Sanchez JC. 2011. A symbiotic brain-machine interface through value-based decision making. PLOS ONE 6(3):e14760 21423797 10.1371/journal.pone.0014760 PMC3056711

[61] Silversmith DB, Abiri R, Hardy NF, Natraj N, Tu-Chan A,.2021.Plug-and-play control of a brain– computer interface through neural map stabilization. Nat. Biotechnol 39(3):326–35 32895549 10.1038/s41587-020-0662-5

[62] Lu HY, Lorenc ES, Zhu H, Kilmarx J, Sulzer J, 2021. Multi-scale neural decoding and analysis. J. Neural Eng 18:045013 10.1088/1741-2552/ac160f PMC8840800 34284369

[63] Nason SR, Vaskov AK, Willsey MS, Welle EJ, An H, 2020. A low-power band of neuronal spiking activity dominated by local single units improves the performance of brain–machine interfaces. Nat. Biomed. Eng 4(10):973–83 32719512 10.1038/s41551-020-0591-0 PMC7982996

[64] Trautmann EM, Stavisky SD, Lahiri S, Ames KC, Kaufman MT, 2019. Accurate estimation of neural population dynamics without spike sorting. Neuron 103(2):292–308.e4 31171448 10.1016/j.neuron.2019.05.003 PMC7002296

[65] Trautmann EM, O’Shea DJ, Sun X, Marshel JH, Crow A,.2021.Dendritic calcium signals in rhesus macaque motor cortex drive an optical brain-computer interface. Nat. Commun 12:3689 34140486 10.1038/s41467-021-23884-5 PMC8211867

[66] Mitani A, Dong M, Komiyama T. 2018. Brain-computer interface with inhibitory neurons reveals subtype-specific strategies. Curr. Biol 28(1):77–83.e4 29249656 10.1016/j.cub.2017.11.035 PMC5760288

[67] Clancy KB, Mrsic-Flogel TD. 2012. The sensory representation of causally controlled objects. Neuron 109(4):677–89.e4 10.1016/j.neuron.2020.12.001 PMC7889580 33357383

[68] Prsa M, Galiñanes GL, Huber D. 2017. Rapid integration of artificial sensory feedback during operant conditioning of motor cortex neurons. Neuron 93(4):929–39.e6 28231470 10.1016/j.neuron.2017.01.023 PMC5330804

[69] Buzsáki G, Anastassiou CA, Koch C.2012.The origin of extracellular fields and currents—EEG,ECoG, LFP and spikes. Nat. Rev. Neurosci 13(6):407–20 22595786 10.1038/nrn3241 PMC4907333

[70] So K, Dangi S, Orsborn AL, Gastpar MC, Carmena JM. 2014. Subject-specific modulation of local field potential spectral power during brain–machine interface control in primates. J. Neural Eng 11:026002 24503623 10.1088/1741-2560/11/2/026002

[71] Flint RD, Wright ZA, Scheid MR, Slutzky MW. 2013. Long term, stable brain machine interface performance using local field potentials and multiunit spikes. J. Neural Eng 10:056005 23918061 10.1088/1741-2560/10/5/056005 PMC4023629

[72] Engelhard B, Ozeri N, Israel Z, Bergman H, Vaadia E. 2013. Inducing γ oscillations and precise spike synchrony by operant conditioning via brain–machine interface. Neuron 77(2):361–75 23352171 10.1016/j.neuron.2012.11.015

[73] Benabid AL, Costecalde T, Eliseyev A, Charvet G, Verney A, 2019. An exoskeleton controlled by an epidural wireless brain–machine interface in a tetraplegic patient: a proof-of-concept demonstration. Lancet Neurol. 18(12):1112–22 31587955 10.1016/S1474-4422(19)30321-7

[74] Hwang EJ, Andersen RA. 2009. Brain control of movement execution onset using local field potentials in posterior parietal cortex. J. Neurosci 29(45):14363–70 19906983 10.1523/JNEUROSCI.2081-09.2009 PMC2805702

[75] Degenhart AD, Bishop WE, Oby ER, Tyler-Kabara EC, Chase SM, 2020. Stabilization of a braincomputer interface via the alignment of low-dimensional spaces of neural activity. Nat. Biomed. Eng 4(7):672–85 32313100 10.1038/s41551-020-0542-9 PMC7822646

[76] Shanechi MM, Orsborn AL, Moorman HG, Gowda S, Dangi S, Carmena JM. 2017. Rapid control and feedback rates enhance neuroprosthetic control. Nat. Commun 8:13825 28059065 10.1038/ncomms13825 PMC5227098

[77] Willett FR, Suminski AJ, Fagg AH, Hatsopoulos NG. 2013. Improving brain–machine interface performance by decoding intended future movements. J. Neural Eng 10:026011 23428966 10.1088/1741-2560/10/2/026011 PMC4019387

[78] Wolpert DM, Flanagan JR. 2001. Motor prediction. Curr. Biol 11(18):R729–32 11566114 10.1016/s0960-9822(01)00432-8

[79] Kao JC, Ryu SI, Shenoy KV. 2017. Leveraging neural dynamics to extend functional lifetime of brainmachine interfaces. Sci. Rep 7:7395 28784984 10.1038/s41598-017-06029-x PMC5547077

[80] Law AJ, Rivlis G, Schieber MH. 2014. Rapid acquisition of novel interface control by small ensembles of arbitrarily selected primary motor cortex neurons. J. Neurophysiol 112(6):1528–48 24920030 10.1152/jn.00373.2013 PMC4137252

[81] Tremblay R, Lee S, Rudy B. 2016. GABAergic interneurons in the neocortex: from cellular properties to circuits. Neuron 91(2):260–92 27477017 10.1016/j.neuron.2016.06.033 PMC4980915

[82] Makino H, Hwang EJ, Hedrick NG, Komiyama T. 2016. Circuit mechanisms of sensorimotor learning. Neuron 92(4):705–21 27883902 10.1016/j.neuron.2016.10.029 PMC5131723

[83] Markowitz DA, Wong YT, Gray CM, Pesaran B. 2011. Optimizing the decoding of movement goals from local field potentials in macaque cortex. J. Neurosci 31(50):18412–22 22171043 10.1523/JNEUROSCI.4165-11.2011 PMC3315593

[84] Garcia-Garcia MG, Marquez-Chin C, Popovic MR. 2020. Operant conditioning of motor cortex neurons reveals neuron-subtype-specific responses in a brain-machine interface task. Sci. Rep 10:19992 33203973 10.1038/s41598-020-77090-2 PMC7672061

[85] Vendrell-Llopis N, Fang C, Qü AJ, Costa RM, Carmena JM. 2022. Diverse operant control of different motor cortex populations during learning. Curr. Biol 32(7):1616–22.e5 35219429 10.1016/j.cub.2022.02.006 PMC9007898

[86] Wu W, Gao Y, Bienenstock E, Donoghue JP, Black MJ. 2006. Bayesian population decoding of motor cortical activity using a Kalman filter. Neural Comput. 18(1):80–118 16354382 10.1162/089976606774841585

[87] Sussillo D, Nuyujukian P, Fan JM, Kao JC, Stavisky SD, 2012. A recurrent neural network for closed-loop intracortical brain–machine interface decoders. J. Neural Eng 9:026027 22427488 10.1088/1741-2560/9/2/026027 PMC3638090

[88] Willsey MS, Nason-Tomaszewski SR, Ensel SR, Temmar H, Mender MJ, 2022. Real-time brain-machine interface in non-human primates achieves high-velocity prosthetic finger movements using a shallow feedforward neural network decoder. Nat. Commun 13:6899 36371498 10.1038/s41467-022-34452-w PMC9653378

[89] Taylor DM, Tillery SIH, Schwartz AB. 2002. Direct cortical control of 3D neuroprosthetic devices. Science 296(5574):1829–32 12052948 10.1126/science.1070291

[90] Orsborn AL, Dangi S, Moorman HG, Carmena JM. 2012. Closed-loop decoder adaptation on intermediate time-scales facilitates rapid BMI performance improvements independent of decoder initialization conditions. IEEE Trans. Neural Syst. Rehabil. Eng 20(4):468–77 22772374 10.1109/TNSRE.2012.2185066

[91] Brandman DM, Hosman T, Saab J, Burkhart MC, Shanahan BE, 2018. Rapid calibration of an intracortical brain-computer interface for people with tetraplegia. J. Neural Eng 15:026007 29363625 10.1088/1741-2552/aa9ee7 PMC5823702

[92] Li Z, O’Doherty JE, Lebedev MA, Nicolelis MAL. 2011. Adaptive decoding for brain-machine interfaces through Bayesian parameter updates. Neural Comput. 23(12):3162–204 21919788 10.1162/NECO_a_00207 PMC3335277

[93] Madduri MM, Burden SA, Orsborn AL. 2021. A game-theoretic model for co-adaptive brain-machine interfaces. In 2021 10th International IEEE/EMBS Conference on Neural Engineering (NER), pp. 327–30. Piscataway, NJ: IEEE

[94] Marathe AR, Taylor DM. 2011. Decoding position, velocity, or goal: Does it matter for brain–machine interfaces? J. Neural Eng 8:025016 21436529 10.1088/1741-2560/8/2/025016 PMC3140465

[95] Golub MD, Yu BM, Chase SM. 2015. Internal models for interpreting neural population activity during sensorimotor control. eLife 4:e10015 26646183 10.7554/eLife.10015 PMC4874779

[96] Gowda S, Orsborn AL, Overduin SA, Moorman HG, Carmena JM. 2014. Designing dynamical properties of brain–machine interfaces to optimize task-specific performance. IEEE Trans. Neural Syst. Rehabil. Eng 22(5):911–20 24760941 10.1109/TNSRE.2014.2309673

[97] Zhang Y, Chase SM. 2015. Recasting brain-machine interface design from a physical control system perspective. J. Comput. Neurosci 39(2):107–18 26142906 10.1007/s10827-015-0566-4 PMC4568020

[98] Blakemore SJ, Frith CD, Wolpert DM. 1999. Spatio-temporal prediction modulates the perception of self-produced stimuli. J. Cogn. Neurosci 11(5):551–59 10511643 10.1162/089892999563607

[99] Eaton RW, Libey T, Fetz EE.2017.Operant conditioning of neural activity in freely behaving monkeys with intracranial reinforcement. J. Neurophysiol 117(3):1112–25 28031396 10.1152/jn.00423.2016 PMC5340878

[100] Suminski AJ, Tkach DC, Fagg AH, Hatsopoulos NG. 2010. Incorporating feedback from multiple sensory modalities enhances brain-machine interface control. J. Neurosci 30(50):16777–87 21159949 10.1523/JNEUROSCI.3967-10.2010 PMC3046069

[101] Johansson RS, Flanagan JR. 2009. Coding and use of tactile signals from the fingertips in object manipulation tasks. Nat. Rev. Neurosci 10(5):345–59 19352402 10.1038/nrn2621

[102] Delhaye BP, Long KH, Bensmaia SJ. 2018. Neural basis of touch and proprioception. Compr. Physiol 8(4):1575–602 30215864 10.1002/cphy.c170033 PMC6330897

[103] Sainburg RL, Poizner H, Ghez C. 1993. Loss of proprioception produces deficits in interjoint coordination. J. Neurophysiol 70(5):2136–47 8294975 10.1152/jn.1993.70.5.2136 PMC10710694

[104] Raspopovic S, Capogrosso M, Petrini FM, Bonizzato M, Rigosa J, 2014. Restoring natural sensory feedback in real-time bidirectional hand prostheses. Sci. Transl. Med 6:222ra19 10.1126/scitranslmed.3006820 24500407

[105] Penfield W, Boldrey E. 1937. Somatic motor and sensory representation in the cerebral cortex of man as studied by electrical stimulation. Brain 60(4):389–443

[106] Histed MH, Ni AM, Maunsell JHR. 2013. Insights into cortical mechanisms of behavior from microstimulation experiments. Prog. Neurobiol 103:115–30 22307059 10.1016/j.pneurobio.2012.01.006 PMC3535686

[107] Cohen MR, Newsome WT. 2004. What electrical microstimulation has revealed about the neural basis of cognition. Curr. Opin. Neurobiol 14(2):169–77 15082321 10.1016/j.conb.2004.03.016

[108] Roche JP, Hansen MR. 2015. On the horizon: cochlear implant technology. Otolaryngol. Clin. N. Am 48(6):1097–116 10.1016/j.otc.2015.07.009 PMC4641792 26443490

[109] Murphey DK, Maunsell JH. 2007. Behavioral detection of electrical microstimulation in different cortical visual areas. Curr. Biol 17(10):862–67 17462895 10.1016/j.cub.2007.03.066 PMC2034326

[110] Doty RW. 1965. Conditioned reflexes elicited by electrical stimulation of the brain in macaques. J. Neurophysiol 28:623–40 14347624 10.1152/jn.1965.28.4.623

[111] Dadarlat MC, O’Doherty JE, Sabes PN. 2015. A learning-based approach to artificial sensory feedback leads to optimal integration. Nat. Neurosci 18(1):138–44 25420067 10.1038/nn.3883 PMC4282864

[112] Tabot GA, Dammann JF, Berg JA, Tenore FV, Boback JL, 2013. Restoring the sense of touch with a prosthetic hand through a brain interface. PNAS 110(45):18279–84 24127595 10.1073/pnas.1221113110 PMC3831459

[113] London BM, Jordan LR, Jackson CR, Miller LE. 2008. Electrical stimulation of the proprioceptive cortex (area 3a) used to instruct a behaving monkey. IEEE Trans. Neural Syst. Rehabil. Eng 16(1):32–36 18303803 10.1109/TNSRE.2007.907544 PMC2586075

[114] O’Doherty JE, Shokur S, Medina LE, Lebedev MA, Nicolelis MA. 2019. Creating a neuroprosthesis for active tactile exploration of textures. PNAS 116(43):21821–27 31591224 10.1073/pnas.1908008116 PMC6815176

[115] Flesher SN, Collinger JL, Foldes ST, Weiss JM, Downey JE, 2016. Intracortical microstimulation of human somatosensory cortex. Sci. Transl. Med 8:361ra141 10.1126/scitranslmed.aaf8083 27738096

[116] Stoney SD, Thompson WD, Asanuma H. 1968. Excitation of pyramidal tract cells by intracortical microstimulation: effective extent of stimulating current. J. Neurophysiol 31(5):659–69 5711137 10.1152/jn.1968.31.5.659

[117] Butovas S, Schwarz C. 2003. Spatiotemporal effects of microstimulation in rat neocortex: a parametric study using multielectrode recordings. J. Neurophysiol 90(5):3024–39 12878710 10.1152/jn.00245.2003

[118] Histed MH, Bonin V, Reid RC. 2009. Direct activation of sparse, distributed populations of cortical neurons by electrical microstimulation. Neuron 63(4):508–22 19709632 10.1016/j.neuron.2009.07.016 PMC2874753

[119] Kumaravelu K, Sombeck J, Miller LE, Bensmaia SJ, Grill WM. 2022. Stoney vs. Histed: quantifying the spatial effects of intracortical microstimulation. Brain Stimul. 15(1):141–51 34861412 10.1016/j.brs.2021.11.015 PMC8816873

[120] Dadarlat MC, Sun Y, Stryker MP. 2019. Widespread activation of awake mouse cortex by electrical stimulation. In 2019 9th International IEEE/EMBS Conference on Neural Engineering (NER), pp. 1113–17. Piscataway, NJ: IEEE 10.1109/NER.2019.8716956 PMC6667156 31363384

[121] Stieger KC, Eles JR, Ludwig KA, Kozai TDY. 2022. Intracortical microstimulation pulse waveform and frequency recruits distinct spatiotemporal patterns of cortical neuron and neuropil activation. J. Neural Eng 19:026024 10.1088/1741-2552/ac5bf5 PMC9171725 35263736

[122] Eles JR, Stieger KC, Kozai TD. 2021. The temporal pattern of intracortical microstimulation pulses elicits distinct temporal and spatial recruitment of cortical neuropil and neurons. J. Neural Eng 18:015001 10.1088/1741-2552/abc29c PMC8167825 33075762

[123] Stieger KC, Eles JR, Ludwig KA, Kozai TD. 2020. In vivo microstimulation with cathodic and anodic asymmetric waveforms modulates spatiotemporal calcium dynamics in cortical neuropil and pyramidal neurons of male mice. J. Neurosci. Res 98(10):2072–95 32592267 10.1002/jnr.24676 PMC8095318

[124] Venkatraman S, Carmena JM. 2009. Behavioral modulation of stimulus-evoked oscillations in barrel cortex of alert rats. Front. Integr. Neurosci 3:10 19521539 10.3389/neuro.07.010.2009 PMC2694660

[125] Trevathan JK, Asp AJ, Nicolai EN, Trevathan JM, Kremer NA, 2021. Calcium imaging in freely moving mice during electrical stimulation of deep brain structures. J. Neural Eng 18:026008 10.1088/1741-2552/abb7a4 PMC8485730 32916665

[126] Kumaravelu K, Tomlinson T, Callier T, Sombeck J, Bensmaia SJ, 2020. A comprehensive model-based framework for optimal design of biomimetic patterns of electrical stimulation for prosthetic sensation. J. Neural Eng 17:046045 32759488 10.1088/1741-2552/abacd8 PMC8559728

[127] Bashivan P, Kar K, DiCarlo JJ. 2019. Neural population control via deep image synthesis. Science 364(6439):eaav9436 31048462 10.1126/science.aav9436

[128] Romo R, Hernández A, Zainos A, Salinas E. 1998. Somatosensory discrimination based on cortical microstimulation. Nature 292:387–90 10.1038/32891 9537321

[129] Leal-Campanario R, Delgado-García JM, Gruart A. 2006. Microstimulation of the somatosensory cortex can substitute for vibrissa stimulation during Pavlovian conditioning. PNAS 103(26):10052–57 16782811 10.1073/pnas.0603584103 PMC1479767

[130] Otto KJ, Rousche PJ, Kipke DR. 2005. Microstimulation in auditory cortex provides a substrate for detailed behaviors. Hear. Res 210(1/2):112–17 16209915 10.1016/j.heares.2005.08.004

[131] Otto KJ, Rousche PJ, Kipke DR. 2005. Cortical microstimulation in auditory cortex of rat elicits bestfrequency dependent behaviors. J. Neural Eng 2(2):42–51 15928411 10.1088/1741-2560/2/2/005

[132] Callier T, Brantly NW, Caravelli A, Bensmaia SJ. 2020. The frequency of cortical microstimulation shapes artificial touch. PNAS 117(2):1191–200 31879342 10.1073/pnas.1916453117 PMC6969512

[133] Butovas S, Schwarz C. 2007. Detection psychophysics of intracortical microstimulation in rat primary somatosensory cortex. Eur. J. Neurosci 25(7):2161–69 17419757 10.1111/j.1460-9568.2007.05449.x

[134] Callier T, Schluter EW, Tabot GA, Miller LE, Tenore FV, Bensmaia SJ. 2015. Long-term stability of sensitivity to intracortical microstimulation of somatosensory cortex. J. Neural Eng 12:056010 26291448 10.1088/1741-2560/12/5/056010

[135] Fitzsimmons NA, Drake W, Hanson TL, Lebedev MA, Nicolelis MAL. 2007. Primate reaching cued by multichannel spatiotemporal cortical microstimulation. J. Neurosci 27(21):5593–602 17522304 10.1523/JNEUROSCI.5297-06.2007 PMC6672750

[136] Doherty JEO, Lebedev MA, Li Z, Nicolelis MAL. 2012. Virtual active touch using randomly patterned intracortical microstimulation. IEEE Trans. Neural Syst. Rehabil. Eng 20(1):85–93 22207642 10.1109/TNSRE.2011.2166807 PMC3590844

[137] Thomson EE, Carra R, Nicolelis MA. 2013. Perceiving invisible light through a somatosensory cortical prosthesis. Nat. Commun 4:1482 23403583 10.1038/ncomms2497 PMC3674834

[138] Richardson AG, Ghenbot Y, Liu X, Hao H, Rinehart C, 2019. Learning active sensing strategies using a sensory brain–machine interface. PNAS 116(35):17509–14 31409713 10.1073/pnas.1909953116 PMC6717311

[139] Sober SJ, Sabes PN. 2003. Multisensory integration during motor planning. J. Neurosci 23(18):6982–92 12904459 10.1523/JNEUROSCI.23-18-06982.2003 PMC6740676

[140] Ernst MO, Banks MS. 2002. Humans integrate visual and haptic information in a statistically optimal fashion. Nature 415(6870):429–33 11807554 10.1038/415429a

[141] Dadarlat MC, Sabes PN. 2016. Encoding and decoding of multi-channel ICMS in macaque somatosensory cortex. IEEE Trans. Hapt 9(4):508–14 10.1109/TOH.2016.2616311 27740497

[142] Risso G, Valle G, Iberite F, Strauss I, Stieglitz T, 2019. Optimal integration of intraneural somatosensory feedback with visual information: a single-case study. Sci. Rep 9:7916 31133637 10.1038/s41598-019-43815-1 PMC6536542

[143] Makino H, Hwang EJ, Hedrick NG, Komiyama T. 2016. Circuit mechanisms of sensorimotor learning. Neuron 92(4):705–21 27883902 10.1016/j.neuron.2016.10.029 PMC5131723

[144] Koralek AC, Jin X, Long JD II, Costa RM, Carmena JM. 2012. Corticostriatal plasticity is necessary for learning intentional neuroprosthetic skills. Nature 483(7389):331–35 22388818 10.1038/nature10845 PMC3477868

[145] Long JD, Carmena JM. 2013. Dynamic changes of rodent somatosensory barrel cortex are correlated with learning a novel conditioned stimulus. J. Neurophysiol 109(10):2585–95 23468389 10.1152/jn.00553.2012

[146] Recanzone GH, Merzenich MM, Dinse HR. 1992. Expansion of the cortical representation of a specific skin field in primary somatosensory cortex by intracortical microstimulation. Cereb. Cortex 2(3):181–96 1511220 10.1093/cercor/2.3.181

[147] Dinse HR, Recanzone GH, Merzenich MM. 1993. Alterations in correlated activity parallel ICMS-induced representational plasticity. NeuroReport 5(2):173–76 8111006 10.1097/00001756-199311180-00020

[148] Fox K, Stryker M. 2017. Integrating Hebbian and homeostatic plasticity: introduction. Philos. Trans. R. Soc. B 372(1715):20160413 10.1098/rstb.2016.0413 PMC5247598 28093560

[149] Jackson A, Mavoori J, Fetz EE. 2006. Long-term motor cortex plasticity induced by an electronic neural implant. Nature 444(7115):56–60 17057705 10.1038/nature05226

[150] Rebesco JM, Stevenson IH, Körding KP, Solla SA, Miller LE. 2010. Rewiring neural interactions by micro-stimulation. Front. Syst. Neurosci 4:39 20838477 10.3389/fnsys.2010.00039 PMC2936935

[151] Rebesco JM, Miller LE. 2011. Enhanced detection threshold for in vivo cortical stimulation produced by Hebbian conditioning. J. Neural Eng 8:016011 21252415 10.1088/1741-2560/8/1/016011 PMC3056083

[152] Recanzone GH, Merzenich MM, Jenkins WM, Grajski KA, Dinse HR. 1992. Topographic reorganization of the hand representation in cortical area 3b of owl monkeys trained in a frequency-discrimination task. J. Neurophysiol 67(5):1031–56 1597696 10.1152/jn.1992.67.5.1031

[153] Ni AM, Maunsell JHR. 2010. Microstimulation reveals limits in detecting different signals from a local cortical region. Curr. Biol 20(9):824–28 20381351 10.1016/j.cub.2010.02.065 PMC2879058

[154] Dalgleish HW, Russell LE, Packer AM, Roth A, Gauld OM, 2020. How many neurons are sufficient for perception of cortical activity? eLife 9:e58889 33103656 10.7554/eLife.58889 PMC7695456

[155] Houweling AR, Brecht M. 2008. Behavioural report of single neuron stimulation in somatosensory cortex. Nature 451(7174):65–68 18094684 10.1038/nature06447

[156] Jeon BB, Fuchs T, Chase SM, Kuhlman SJ. 2022. Existing function in primary visual cortex is not perturbed by new skill acquisition of a non-matched sensory task. Nat. Commun 13:3638 35752622 10.1038/s41467-022-31440-y PMC9233699

[157] Salzman DC, Britten KH, Newsome WT. 1990. Cortical microstimulation influences judgements of motion direction. Nature 346:174–77 2366872 10.1038/346174a0

[158] Romo R, Hernández A, Zainos A, Brody CD, Lemus L.2000.Sensing without touching: psychophysical performance based on cortical microstimulation. Neuron 26(1):273–78 10798410 10.1016/s0896-6273(00)81156-3

[159] Hughes CL, Flesher SN, Weiss JM, Boninger M, Collinger JL, Gaunt RA. 2021. Perception of microstimulation frequency in human somatosensory cortex. eLife 10:e65128 34313221 10.7554/eLife.65128 PMC8376245

[160] Salas MA, Bashford L, Kellis S, Jafari M, Jo H, 2018. Proprioceptive and cutaneous sensations in humans elicited by intracortical microstimulation. eLife 7:e32904 29633714 10.7554/eLife.32904 PMC5896877

[161] Heming E, Sanden A, Kiss ZHT. 2010. Designing a somatosensory neural prosthesis: percepts evoked by different patterns of thalamic stimulation. J. Neural Eng 7:064001 21084731 10.1088/1741-2560/7/6/064001

[162] D’Anna E, Petrini FM, Artoni F, Popovic I, Simanić I, 2017. A somatotopic bidirectional hand prosthesis with transcutaneous electrical nerve stimulation based sensory feedback. Sci. Rep 7:10930 28883640 10.1038/s41598-017-11306-w PMC5589952

